# Factors influencing decision of general practitioners and managers to train and employ a nurse practitioner or physician assistant in primary care: a qualitative study

**DOI:** 10.1186/s12875-017-0587-3

**Published:** 2017-02-07

**Authors:** Mieke van der Biezen, Emmy Derckx, Michel Wensing, Miranda Laurant

**Affiliations:** 1 Radboud university medical center, Radboud Institute for Health Sciences, IQ healthcare, Scientific Center for Quality of Healthcare, P.O. Box 910, Nijmegen, 6500 HB The Netherlands; 2 Foundation for Development of Quality Care in General Practice, Tilburgseweg-West 100, Eindhoven, 5652 NP The Netherlands; 3 Department of General Practice and Health Services Research, Heidelberg University, INF- Marsilius Arkaden, Im Neuenheimer Feld 130.3, Heidelberg, 69120 Germany; 4 HAN University of Applied Sciences, Faculty of Health and Social Studies, P.O. Box 6960, Nijmegen, 6503 GL The Netherlands

**Keywords:** Substitution, Supplementation, Skill mix, General practitioner, Nurse practitioner, Physician assistant, Primary care, Implementation

## Abstract

**Background:**

Due to the increasing demand on primary care, it is not only debated whether there are enough general practitioners (GPs) to comply with these demands but also whether specific tasks can be performed by other care providers. Although changing the workforce skill mix care by employing Physician Assistants (PAs) and Nurse Practitioners (NPs) has proven to be both effective and safe, the implementation of those professionals differs widely between and within countries. To support policy making regarding PAs/NPs in primary care, the aim of this study is to provide insight into factors influencing the decision of GPs and managers to train and employ a PA/NP within their organisation.

**Methods:**

A qualitative study was conducted in 2014 in which 7 managers of out-of-hours primary care services and 32 GPs who owned a general practice were interviewed. Three main topic areas were covered in the interviews: the decision-making process in the organisation, considerations and arguments to train and employ a PA/NP, and the tasks and responsibilities of a PA/NP.

**Results:**

Employment of PAs/NPs in out-of-hours services was intended to substitute care for minor ailments in order to decrease GPs’ caseload or to increase service capacity. Mangers formulated long-term planning and role definitions when changing workforce skill mix. Lastly, out-of-hours services experienced difficulties with creating team support among their members regarding the employment of PAs/NPs.

In general practices during office hours, GPs indented both substitution and supplementation for minor ailments and/or target populations through changing the skill mix. Supplementation was aimed at improving quality of care and extending the range of services to patients. The decision-making in general practices was accompanied with little planning and role definition. The willingness to employ PAs/NPs was highly influenced by an employees’ motivation to start the master’s programme and GPs’ prior experience with PAs/NPs. Knowledge about the PA/NP profession and legislations was often lacking.

**Conclusions:**

Role standardisations, long-term political planning and support from professional associations are needed to support policy makers in implementing skill mix in primary care.

**Electronic supplementary material:**

The online version of this article (doi:10.1186/s12875-017-0587-3) contains supplementary material, which is available to authorized users.

## Background

With an aging population, more patients with chronic complaints and reforms that shift care from hospitals to the community, the pressure on primary care is high [1–3]. It is not only debated whether there are enough general practitioners (GPs) to comply these increasing demands but also whether specific tasks can be transferred to other care providers. Changing the healthcare workforce skill mix has been applied to improve effectiveness and efficiency of healthcare [4]. Around the world, physician assistants and nurse practitioners (PAs/NPs) have been involved in primary care. Although definitions, education and legislation of PAs/NPs differ per country, there is a common ground that PAs/NPs are trained to diagnose and treat defined patient groups (semi-) independently or under physician supervision [2, 3, 5]. Research has shown that PAs/NPs can substitute for GPs on a wide range of patient care tasks, resulting in at least comparable outcomes to those of GPs and higher patient satisfaction [1, 2, 6]. In addition, PAs/NPs can be involved in specific complementary roles such as preventive care or home visits [2, 7, 8]. Nevertheless, the implementation of PAs/NPs differs between and within countries [9–12]. In the Netherlands, most PAs/NPs work in hospital settings and their implementation in primary care is still at a pioneering stage [13, 14]. The number of PAs/NPs in relation to GPs (headcount) is approximately 1 PA and 2 NPs per 100 GPs [15, 16].

PAs/NPs have worked in the Netherlands since 2001. The education of PAs/NPs in the Netherlands includes a master’s programme of respectively 2.5 and 2 years at universities of applied sciences [10]. Their education incorporates a dual work-education model, meaning that students are employed within a practice and receive salary [17]. Although PAs’ education is based on a medical model and NPs’ education on a nursing model, the position of PAs and NPs in primary care is often regarded as interchangeable [18–20]. In the Netherlands both care providers are allowed to prescribe drugs and perform certain preserved procedures related to diagnosis and treatment independently [10, 21]. However, there is still a lack of clarification regarding their role and value in primary care; a situation observed internationally [2, 22, 23]. So far, the Dutch College of General Practitioners (NHG) and National Association of General Practitioners (LHV) have not proposed a role for PAs/NPs in the basic primary care team in general practice [10].

Currently there is limited insight into the reasons to employ, or not to employ, a PA/NP in primary care. Evidence from European countries is lacking or outdated [4]. To support policy making, more evidence is needed about the reasons and perspectives of GPs and managers to train and employ PAs/NPs in primary care.

## Methods

### Study aim and design

A qualitative study to provide insight into factors influencing the decision of GPs and managers to train and employ a PA/NP within their organisation.

### Setting & cohort

**Fig. 1:**
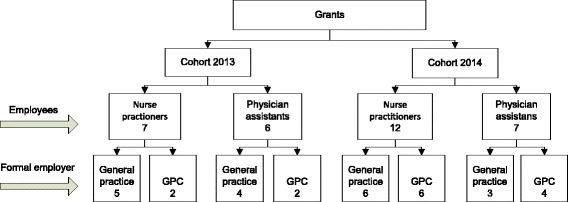
Overview study cohort

In the Netherlands, GPs are a patient’s first point of contact and the 24/7 gatekeepers for secondary care. During office hours, the majority of GPs work in small-scale general practices (eighty percent are duo or solo practices) [24, 25]. To deliver out-of-hours care, GPs from a region are organised in general practitioner cooperatives (GPCs). At those GPCs 40 to 250 GPs take turns on being on duty to take care of populations ranging from 100,000 to 500,000 citizens [26, 27]. In 2013 a project was initiated in the Netherlands that offered both general practices and GPCs additional financial support to train a PA/NP within their organisation. PAs/NPs who were formally employed by GPCs were trained during office hours in general practices in the region. Those organisations whose application for financial support was granted were included in the study cohorts. There was no further selection of participants and all organisations were included. The 2013 cohort included 13 PAs/NPs and the 2014 cohort included 19 PAs/NPs who were either formally employed by a general practice or a GPC (see Fig. 1). We included one GP from all general practices where the PAs/NPs received their training during office hours. In addition, in the case that the PA/NP was formally employed at a GPC, we also included a manager of that GPC. Some GPCs employed more than one PA/NP.

### Data collection

Data were collected between September 2014 and January 2015. As there was a lack of previous knowledge and the topic of PAs/NPs in primary care might be considered controversial, individual semi-structured interviews were chosen to obtain in-depth information about the experiences of the participants. Interviews were conducted either face-to-face at the practice site or by telephone. Three main topic areas were covered in the interviews: determinants of the decision-making process, considerations and arguments to train and employ a PA/NP, and PAs’/NPs’ tasks and responsibilities. Each topic area included 3 or 4 open-ended questions to encourage participants to discuss their perspectives and considerations. The interview guide was developed by the primary researcher (MB) with guidance from the co-authors (ML, ED). An additional file shows the interview guide in more detail [see Additional file 1]. The interviews were conducted by the primary researcher; a health scientist trained in qualitative research methods. Interviews were audiotaped and then transcribed verbatim.

### Data analysis

The transcribed interviews were analysed using content analysis, a qualitative research method to systematically organise data into a structured format [28]. First, two researchers (MB, IM) independently coded the transcripts using an inductive approach. Coding was done with constant comparison of interpretations and the generated list of codes was developed into a shared codebook. During face-to-face meetings the codes of the two researchers were discussed until consensus was reached. Next, coding was followed by a collaborative interpretation in which data was mapped into themes and subthemes. Data was declared to have reached saturation when no new themes were emerging. Lastly, these (sub) themes were discussed for framing of the results and the final refinement of the codes and themes was done by the research team (MB, ML). Atlas.ti software V.7.1.5. was used to facilitate the coding process.

## Results

### Study population

A total of 32 PAs/NPs started their training in general practices in September 2013 or in September 2014. All GPs owning those practices participated in the interviews. Thirteen of the 32 PAs/NPs were formally employed by a GPC. Therefore, a manager from each GPC was interviewed in addition to the interviews with the GPs. Table 1 provides an overview of the interviewees and there were no refusals to participate. General practices and GPCs were situated across the Netherlands. The first six interviews took place at the practice site and all others were conducted by telephone. The mean duration of the interviews was 51 min (SD 12.71). Only 5 GPs had experience working with a PA/NP in their practice prior to the project.

**Table 1:** Characteristics of interviewees (*N =* 39)

General practitioner solo practice	13	(33.3%)
General practitioner duo practice	8	(20.5%)
General practitioner group practice	11	(28.2%)
Manager general practitioner cooperative	7	(17.9%)
Male	18	(46.2%)
Female	21	(53.8%)
Mean age GPs (years)	48	(SD 8.6)

The analysis resulted in three themes from which 11 categories emerged (see Table 2).

**Table 2:** Themes and categories influencing the decision of GPs and managers to train and employ a PA/NP

Theme I. Reasons to employ a PA/NP	
Substitution of care	
Quality improvement	
New/ additional services	
Theme II. Influencing factors	
Organisational factors	
Factors regarding professional relations	
Factors regarding GPs’ workload and job satisfaction	
Experience with the PA/NP profession	
Vision about the PA/NP profession	
Insecurities regarding the PA/NP profession	
Theme III. PAs’/NPs’ tasks and responsibilities	
Direct patient care	
Indirect patient care	

### Theme I. Reasons to employ a PA/NP

Three categories emerged from this theme explaining why GPs or GPCs employ a PA/NP: (1) substitution of care; (2) quality improvement; and (3) new/ additional services.

#### Substitution of care

The main reason for GPs and GPCs to employ a PA/NP was substitution of care. They wanted a professional who is capable and authorised to treat patients independently and take over surgery hours from GPs. This is a response to the increasing workload for GPs due to changes in patient population (e.g. ageing populations, multi-morbidity) and changes in organisation of healthcare (e.g. task shifts from hospital to primary care and an increase in demand to participate in community projects). Some GPs wanted to employ the PA/NP in order to replace a GP, to expand the number of patients in their practice or to create job opportunities for their own professional development (e.g. focussing on more complex patients, more time for study or ancillary activities). Only one GP indicated a shortage of GPs as a reason to employ a PA/NP.

*“The intention is to get more time for the increase in complex problems we have to deal with in the near future. This is the start of an evolution: ageing, substitution, tasks shifts from hospital care to primary care. I expect that GPs will get larger practices and cannot comply with the demand for care without support.”*

*(GP group practice, employing a PA)*

For the GPCs an important determining factor was the opening of an emergency care access point (ECAP) for out-of-hours emergency care. This new model is expected to increase the number of consultations at the GPC resulting in an increase in number of shifts per GP.

*“With the opening of a new ECAP we expect an increase in workload that cannot be answered by the GPs in the region. Therefore, we initiated to work with NPs to meet the increase in patients so that GPs can focus on the complex patients.”*

*(Manager GPC, employing an NP)*

#### Quality improvement

By employing a PA/NP most GPs expected to improve the quality of care provided within their practice. Under quality improvement they mentioned: more continuity of care compared to employing a young doctor (often young doctors start their own practice sooner or later); schedule more time for complex patients; more monitoring of target populations and less waiting time for patients with minor ailments. GPs who employed an NP considered a nursing view complimentary to the medical view of the GP. GPs expected NPs to pursue a mix of cure and care and have better collaboration with other care providers in primary care like community nurses. Other quality improvements are related to new additional services.

*“I am experienced with GPs employed by another GP. Yet the downside is that they will leave as soon as they can start their own practice, which causes disturbance among the patients.”*

*(GP solo practice, employing a PA)*

*“Looking at the NPs at the GPC, I notice they comply even more to guidelines and bring a nursing view into their considerations that is of added value for patient care.”*

*(GP group practice, employing an NP)*

#### New/ additional services

Some GPs considered the employment of a PA/NP an opportunity to implement new services within their practices. Most often these services reflect the current policies of the Ministry of Health, Welfare and Sport, health insurance companies and shifts in populations to treat. For example, increasing monitoring of elderly or participating in community projects for preventive care. Some GPs wanted an expansion in care settings for patients such as offering surgery hours in nursing homes or providing hospital care (e.g. diagnostics) in their practices.

*“There are machines to measure the COPD condition, which is something that can easily be delegated to a PA. That would be an improvement in patient care and a lower burden for patients who otherwise have to go to the city.”*

*(GP solo practice, employing a PA)*

Quality improvement and new/additional services were not mentioned by GPCs as a reason to employ PAs/NPs.

### Theme II. Influencing factors decision-making process

There were several factors influencing the decision to employ a PA/NP. Six categories emerged around this theme: (1) Organisational factors; (2) Factors regarding professional relations; (3) Factors regarding GPs’ workload and job satisfaction; (4) GPs’ experience with the PA/NP profession; (5) Vision of the PA/NP profession; and (6) Insecurities regarding the PA/NP profession.

#### Organisational factors

For some GPs financial certainty was an important factor and they let an accountant calculate the financial impact of employing a PA/NP. As a consequence of the implementation of the PA/NP, support staff from practices without prior experience with PAs/NPs needed extra guidance in the triage of patients to the right care provider. Lastly, having a sufficient number of surgery rooms was a precondition for all GPs.

*“When you compare practices with GPs only with practices with GPs and NPs, you need a shift in the organisation, for example triage nurses need to decide which patient has to be treated by which care provider.”*

*(GP group practice, employing an NP)*

For GPCs, the preparation of implementing a new discipline within the organisation took a lot of effort and had an impact on several departments (e.g. human resources and finances), site managers and support staff. A main issue for GPCs was the fact they offer care out-of-hours. In order to maintain a balanced work-private life, they could only offer small contracts with a maximum of eight hours per week. They therefore rely on general practices’ willingness to offer more contract hours to the PA/NP during office hours. However, finding those practices was difficult. GPs often missed the experience and knowledge about PAs/NPs or they criticised their role in primary care. When GPs were positive, a shortage of surgery rooms or financial factors negatively influenced the implementation. Lastly, some GPCs expressed difficulties recruiting PAs/NPs with the proper preliminary training and/or experience, or with a supportive home situation.

*“It is very hard to find GPs who are willing to offer work during office hours. Often the preconditions cannot be met; not enough surgery rooms, colleagues are not supportive, finances don’t fully cover, ‘we already have so many employees in our practice’. We have heard all of these arguments before.”*

*(Manager GPC, employing an NP)*

#### Factors regarding professional relations

For most GPs the primary motive to employ a PA/NP was either a willingness to meet the concerns of GPCs by offering the PA/NP a job opportunity during office hours, or maintaining an appreciated team member. Most often this team member was a practice nurse who already worked within the practice and had the ambition to expand his or her nursing practice by becoming a PA or NP. GPs wanted to meet this ambition in order to keep this employee for their practice. Support among their staff was for almost all GPs a requirement to employ a PA/NP. Another precondition was sometimes the collaboration with another practice.

*“The PA training was the choice of the PA herself, whereas for me an important factor to approve the training was losing her as an employee if I wouldn’t have provided her the opportunity.”*

*(GP solo practice, employing a PA)*

For GPCs decisions are made by a management team in consultation with the members council (i.e. GPs who own practices in the region). Creating a support base for the employment of PAs/NPs with their members was therefore of great importance and very time consuming. GPCs experienced that the negative viewpoint of the professional associations made GPs reluctant to employ a PA/NP in their practices during office hours.

*“You can say, there is no support from GPs’ professional associations for the position of PAs in general practices. That makes GPs hesitant to include this profession within their own ranks.”*

*(Manager GPC, employing a PA)*

#### Factors regarding GPs’ workload and job satisfaction

As a consequence of the PA/NP treating the less complex patients, all GPs expected a difference in their own caseload. While some GPs considered this an opportunity for their own professional growth and enhancing job satisfaction, others feared a more complex caseload. This included a fear of losing routine in treating minor ailments or an increased work pressure due to more complex complaints during surgery hours. Some GPs expected difficulties in taking proper responsibility for their patients and feared missing out on things in case the PA/NP takes over patient care. Detailed knowledge about the Dutch legislation regarding PAs/NPs was often lacking.

*“Look, you delegate a great part of the care. That means you partly lose sight. But the same would happen were you to employ another GP.”*

*(GP solo practice, employing an NP)*

*“Maybe a disadvantage is a transformation in GPs’ surgery hours in case the number of low complexity problems decreases and the majority of problems become highly complex.”*

*(GP group practice, employing an NP)*

#### GPs’ experience with the PA/NP profession

Some GPs previously worked together with a PA/NP, either at a GPC or in a foreign country, which positively influenced their decision to employ a PA/NP. Also positive experiences of colleagues influenced the decision. However, due to the small number of PAs/NPs in primary care in the Netherlands not many GPs had experience with them or knew a colleague with a PA/NP and they made little effort to to get in contact with any colleague.

*“We experienced working together with NPs at the GPC and my colleague and I were very satisfied with their functioning. That made us curious how it would work out in our practice.”*

*(GP group practice, employing an NP)*

*“I am always eager to innovate, so I thought “why not?”. This was also because I had heard from a colleague that they are really happy with their physician assistant.”*

*(GP solo practice, employing an NP)*

#### Vision of the PA/NP profession

In general, GPs had a lack of knowledge regarding the differences between the PA and the NP professions. They often did not know the differences in education and scope of practice. The choice to employ either a PA or an NP was mostly the training preference of the applicant. Some GPs had let themselves be informed by the Foundation for Development of Quality Care in General Practice and one GP made his decision based on scientific literature. GPs considered the PA to be more medically educated and the NP to be more connected with care in general. There were also inaccuracies expressed, for example the belief that a nurse could not apply for a PA training. GPs who employed a PA did not express much preference regarding previous professional experience and preliminary training. The majority of GPs employing an NP considered a nursing education required to treat the broad spectrum of complaints in primary care. Moreover, they often considered nursing experience in several clinical hospital departments to be favourable.

In many cases curiosity played a role in the decision to employ a PA/NP and preparation of the implementation was often lacking. Most GPs did not have a clear insight in the role of PAs/NPs in other primary care practices or in the exact curriculum of the training. A clear long-term vision about the role of the PA/NP in their practice was often not expressed.

*“I should really revisit the differences between a PA and an NP, because for me those definitions somewhat overlap. So, no, we didn’t really discuss or look at which professional we would employ.”*

*(GP group practice, employing an NP)*

*“We started without good preparation or a detailed plan about what we exactly wanted to achieve in the long term. How do we want to shape our practice? We weren’t sufficiently aware about how the profession in general practices works.”*

*(GP group practice, employing an NP)*

*“The single-handed GP like it used to be is something that is slowly changing and completely disappearing. Now you are building a team within general practices. Maybe it should be called primary care team, in which many professionals collaborate in one centre, with the common goal of improving healthcare in the broadest sense. The GP has a role, just as NPs do. It’s the team that puts it together.”*

*(GP group practice, employing an NP)*

In contrast to GPs, GPCs made more informed decisions regarding the employment of a PA or an NP. This was often based on the curriculum of the training or preferences of the GPs in their region. They often formulated a long-term vision of the PA's/NP's role within their organisation.

#### Insecurities regarding the PA/NP profession

GPs without prior experience working with PAs/NPs expressed uncertainties about their own profession, in particular that an actual decrease in their workload is not guaranteed. Almost all GPs expressed uncertainties about the future in terms of the financial reimbursement of primary care and about political decisions regarding legislation and scope of practice of PAs/NPs.

Both GPs and GPCs felt that political and financial uncertainties made it difficult to formulate long-term organisation planning. Moreover, investments in the training and employment of a PA/NP cause uncertainties because there is no guarantee that these will be paid back.

*“Rules keep on changing during the play. Planning ahead and ensuring a financial base is difficult as there are no certainties in general practice.”*

*(GP duo practice, employing an NP)*

### Theme III. PAs’/NPs’ tasks and responsibilities

#### Direct patient care

Most GPs had not formulated an exact role description including the scope of PA’s/NP’s practice and GPs differed considerably in their ideas about the task of the PA/NP within their practice. Some GPs wanted the PA/NP to serve certain target populations, while others wanted the PA/NP to serve a broad range of patient complaints. Different views were expressed as to whether or not PAs/NPs should treat chronic patients, acute problems, palliative care, gynaecology complaints and care for elderly or young children. In general, there were some complaints that all GPs agreed were suitable for PA/NP practice. These minor ailments included: dermatology, ear nose and throat complaints, musculoskeletal system and influenza. In addition, preventive tasks like social home visits and postoperative consultations were considered suitable. GPs considered complaints to be suitable when they are not life threatening and if there is a low impact when something goes wrong. The role description was based on the curriculum of PA’s/NP’s training, experience of the PA/NP, the number of patients having those complaints, and being straightforward for triage.

*“We wanted a professional who could take over parts of our job. For example, we were thinking about ear complaints, children with fever, abdominal pain, urological infections, et cetera. So, well defined areas that are straightforward for triage.”*

*(GP duo practice, employing a PA)*

*“In palliative care, a lot of tasks are not medical based, but rather nurse based. We intend the NP to be responsible for organising everything at home when a patient is discharged from the hospital; keeping everyone informed about agreements and having insight in expected complications.”*

*(GP group practice, employing an NP)*

In contrast to the general practices, the GPCs formulated a role description for the PA/NP for the shifts at the GPC. This role description included a number of complaints that were excluded from PA/NP care, all other complaints were considered within PAs’/NPs’ scope of practice. At most GPCs those complaints were: abdominal pain, cardiological, neurological and psychiatric complaints and children younger than 1 year old. GPs who trained the PAs/NPs who were employed by the GPC, were free to make decisions on PAs’/NPs’ tasks within their practices during office hours. In practice, they usually used the same role description as the GPC.

#### Indirect or non-patient related tasks

In general, both GPs and GPCs had not considered indirect or non-patient related tasks much. They wanted the PA/NP to focus on direct patient care first. Indirect or non-patient related tasks were likely to be considered when the PA/NP would be more experienced. Tasks considered suitable were: meetings with other primary care professionals, coordination of elderly care, developing protocols and training support staff.

*“Of course the PA can participate in projects like quality improvement, practice accreditation, but we don’t really know exactly yet, we’ll just see.”*

*(GP group practice, employing a PA)*

## Discussion

Employing PAs/NPs in general practices requires role revision. The literature describes two conceptually different approaches to role revision. The first is to employ PAs/NPs as substitutes for GPs, the second as supplements [3]. The GPCs in the current study primarily aim at substitution of GPs within teams offering out-of-hours care. That means PAs/NPs provide the same services as GPs in order to decrease GPs’ caseload or increase service capacity as a reaction to the increased workload due to the opening of ECAPs. General practices on the other hand, do not just aim at substitution but also at supplementing GPs. The aim of supplementing is to improve quality of care and extend the range of services to patients. By extending the range of services GPs are able to meet the increase in tasks and patient groups in primary care [3]. An extension in services is also considered a quality improvement. In addition, GPs consider the added value of PAs/NPs as a quality improvement. The services provided by PAs/NPs are considered less medically focussed and instead, often based on their orientation in the nursing discipline, have a more holistic focus [22]. Both GPCs and GPs consider care for patients with minor ailments to be within a broad spectrum suitable for PA/NP care. Some GPs also employ PAs/NPs for certain target populations either as substitute or supplementary to GPs. A study about PAs as case managers for geriatric conditions, suggests that incorporating PAs in supplemental roles for target populations can increase quality of care for previously underdiagnosed and undertreated conditions [2].

According to Contandriopoulos et al. [29] several themes are important for an effective model for integrating new roles in primary healthcare teams. These themes include planning, role definition, practice model, collaboration and team support. The GPCs in the current study put a lot of effort into advance planning. They developed a comprehensive plan and formalised the role of the PA/NP in writing. Moreover, they expressed a clear vision on how the practice model of PAs/NPs in teams together with GPs provides out-of-hours care. In larger organisations like GPCs this clear role formalisation is especially important [29]. Although GPCs put a lot of effort into creating support among their members, due to the large number of members, not all members were supportive about implementing PAs/NPs. As a result, GPCs experience uncertainties about their financial investment in PAs/NPs because GPCs highly depend on general practices to provide the PAs/NPs employment during office hours. Influencing factors, also shown in the literature, for the negative viewpoint among GPs are the lack of support from GPs’ professional associations or not being convinced of the added value of PAs/NPs in primary care [30–32].

The implementation process in general practices differs from GPCs. International studies have shown that inadequate planning is very common for general practices [33, 34]. General practices are understood as complex responsive processes of humans relating to each other. GPs do not usually develop a blueprint for the change that comes with the PA/NP role in their practice. However decisions are not simply random either [35]. As Contandriopoulos et al. [29] describe “*integrating NPs into primary care teams is likely to be a dynamic, complex, and messy process*”. Decisions emerge in the interplay of intentions, communicative gestures and responses, power-relating and values-based choices and actions of the partners, practice staff and policy makers in a range of areas [35]. In the current study the determining factors to employ a PA/NP were often: 1. an employee who wanted to start the PA or NP master’s programme, or 2. GPs wanting to support the GPC by providing employment and training to the PA/NP during office hours. Just as in previous studies, most GPs expressed that their willingness to employ a PA/NP was influenced by prior knowledge or working experience with PAs/NPs [4, 30, 36]. However, knowledge about the PA/NP profession, legislation and role definitions in general practice is often lacking. Just as in other studies there are preconceived notions about PAs’/NPs’ roles and training and the difference between a PA and an NP is not clear [30, 37, 38]. GPs from practices without prior experience working with PAs/NPs felt hesitant about the changes in caseload and whether their workload actually decreases when they employ a PA/NP. Their hesitance can be supported by literature showing that employing PAs/NPs does not always result in a reduced workload, especially when the GP continues to provide the care that has been substituted. Moreover, with the employment of PAs/NPs the complexity of patients for GPs increases [2, 4, 39]. The development of the practice model is, as previously observed in other studies, often emerging through trial and error. GPs base their vision on the needs of their patient population and the experience and preferences of themselves and the PAs/NPs [2, 29]. In addition, the roles of the PAs/NPs are often not well defined. Although many studies indicate an inappropriate and incoherent definition of the PA/NP scope of practice as a big obstacle for PA/NP integration, this does not necessarily mean that roles should be formalised in writing (which is especially the case for small practices). Instead role formalisation should preferably be flexible and allow team members’ practices to evolve [29]. Lastly, GPs rarely expressed problems in collaboration and creating team support regarding the employment of a PA/NP.

As there is a lack of international defined role standards and clarity, each organisation formulates its own scope of practice. International standardisation of PA/NP roles can resolve some of the confusion perceived by other professionals and would enable PAs/NPs to practice to their potential [12, 30, 40]. Moreover, like the GPs in the current study indicate, it is important that political decisions and finances regarding the PA/NP profession in the future are clear and transparent [23, 41].

### Strengths and limitations

The current study has several limitations. First, only GPs and GPCs who had already decided to train and employ PAs/NPs were included in the study. Therefore a comprehensive overview in barriers perceived by GPs and GPCs who decide not to employ PAs/NPs cannot be given. Moreover, the role of the PA/NP in primary care is still at a pioneering stage in the Netherlands and the implementation might be different when PAs/NPs are more widespread. However, the stage of PA/NP implementation differs a lot between countries and so the current study gives a broad overview about implementation by the early adaptors in the field.

Not all findings were found across all practices or participants. The main variation was found between GPCs and general practices. We did not find variation between practices employing a PA or an NP, confirming that PAs and NPs are often regarded as interchangeable in primary care [18, 19]. Some outcomes, such as how a change in caseload for GPs was perceived, differed considerably between individuals. Lastly, there were few practices that had prior experience with a PA/NP within their practice. As most outcomes did not differ for practices without prior experience then these practices were not treated as a separate subgroup.

A strength of the current study is the large number of interviews. Participants were from a broad geographical area and the variation in type of practices reflects the distribution of practices in the Netherlands [42]. Second, interviews were conducted in a semi-structured manner with open coding, which allowed researchers to preserve all information. A potential limitation might be that the setting of interviews was not identical. The majority of the interviews were conducted by telephone, which allowed the researchers to include practices across the entire country. We did not experience differences between the face-to-face and telephone interviews that could have caused data loss or distortion and this is supported by literature on qualitative research interviews [43].

It should be noted that the current study indicates several themes that influenced implementation of the PA/NP in Dutch general practice. It is difficult to translate these to county-specific characteristics due to differences in PA/NP autonomy, PAs’/NPs’ level of education and differences in healthcare systems [17, 31, 44]. Moreover, there is a lack of international and national role standardisation and the implementation of PAs/NPs differs between and within countries [9–12]. In contrast to countries like the United Kingdom, United States, Canada and Australia, the PA/NP role in primary care is relatively new in many countries (including the Netherlands) or non-existent [22, 44]. However, countries with large numbers of PAs/NPs in primary care also struggle with role clarifications and authorities [22]. Further research is therefore needed about the roles and the implementation of PAs/NPs from different countries [5, 11].

## Conclusion

The current study considerably improves our understanding of the factors influencing the decision to employ PAs/NPs in primary care. GPCs intended substitution of care of minor ailments and formulated long-term planning and role definitions. They experienced difficulties with creating team support. In general practices, GPs indented both substitution and supplementation of GPs for minor ailments and/or target populations. The decision-making in general practices was a dynamic process with less planning and role definition. Although roles should be able to evolve over time, a comprehensive long-term practice planning is advisable. Role standardisations, long-term political planning and support from professional associations are needed to support policy makers in implementing PAs/NPs in primary care.

## Additional file

Additional file 1: ‘Cohort 2013 and 2014 – Interview guide general practitioners and managers’. Details of the interview guide translated in English, original language Dutch. (PDF 141 kb)

## Abbreviations

GP: General practitioner
GPC: General practitioners cooperative
NP: Nurse practitioner
PA: Physician assistant
