# Supplementary material for: Factors influencing decision of general practitioners and managers to train and employ a nurse practitioner or physician assistant in primary care: a qualitative study
Source: BMC Fam Pract. 2017 Feb 7;18:16. doi: 10.1186/s12875-017-0587-3 (PMC5297134; doi:10.1186/s12875-017-0587-3)
Supplement: Additional file 1: — ‘Cohort 2013 and 2014 – Interview guide general practitioners and managers’. Details of the interview guide translated in English, original language Dutch. (PDF 141 kb) [file 12875_2017_587_MOESM1_ESM.pdf]

Interview guide [originally in Dutch]

Evaluation "regeling versterking opleiding VSPA huisartsenzorg"

Cohort 2013 and 2014 – Interview guide general practitioners and managers

Name interviewer: …………………………

Date interview: …………………………

Duration interview: …………………………

Number participant: …………………………

Instructions

Each topic starts with an overall question. After reading the question, focus on the interviewee. After the first question the interviewee gets time to respond. Supplementary questions are indicated in the guide by an arrow. You don't necessary have to ask all supplementary questions, depending how the interview develops.

Several non-verbal communication skills are important during the interview[1]

- Create a comfortable environment including transparency, trust and space, connect for example by adapting to the language and information level of the interviewee
- Listen active
- Don't be afraid for silence. The interviewee will be encouraged to tell more
- Be aware of your role as interviewer, reflect on yourself and take notes
- Don't assume you are on the same page. Check this by summarizing
- Ask clarification in case of contradictions
- Strategic use of expressing misunderstanding by asking for elaboration; "how is that going?", "Can you tell more?".

Encourage the interviewee:

- Summarize what has been said: "If I understand it correctly"
- Repeat what has been said or use silences
- Reformulate the question
- Ask for details; "How does X work?"
- Repeat the aim of the interview
- Use indirect questions: action question (what will you do when...), depersonalised questions (the newspaper said...), hypothetical questions (imagine X would happen...)

Structuring the interview:

- Summarize
- Ask again for unfinished topics
- Checking of insinuations

[1] Wester, F. (1995) Strategieën voor kwalitatief onderzoek. Coutinho, Bussum

Introduction of interviewer

- Introduce yourself [name], [profession], [department]
- Explain aim interview
- Indicate time frame interview
- Explain the privacy of the interviewee
- Ask consent to audio tape the interview

Decision to train and employ a PA/NP

1. *We are interested in the decision-making process of training and employing a PA/NP in your organisation*
   → How did the decision take place in your organisation?
   → Who were involved with the decision?
   → What were the reasons to employ a PA/NP, instead of a PA or NP (depending whether they employ a PA or NP) or another GP?
   → How did your organisation prepare itself for the employment of the PA/NP?

2. What were for you the reasons to employ a PA/NP?
   → What was the aim to train a PA/NP?
   → What are for you the expected benefits of employing a PA/NP?
   → What are for you expected disadvantages to employ a PA/NP?

3. What will be the role of the PA/NP within your organisation?
   → What will be patient related tasks? Why those?
   → What will be indirect patient related tasks? Why those?
   → What will be non patient related tasks? Why those?
